# A longitudinal analysis of the completeness of maternal HIV testing, including repeat testing in Cape Town, South Africa

**DOI:** 10.1002/jia2.25441

**Published:** 2020-01-29

**Authors:** Shani de Beer, Emma Kalk, Max Kroon, Andrew Boulle, Meg Osler, Jonathan Euvrard, Venessa Timmerman, Mary‐Ann Davies

**Affiliations:** ^1^ Centre for Infectious Disease Epidemiology & Research School of Public Health and Family Medicine University of Cape Town Cape Town South Africa; ^2^ Department of Paediatrics Mowbray Maternity Hospital University of Cape Town Cape Town South Africa; ^3^ Health Impact Assessment Provincial Government of the Western Cape Cape Town South Africa

**Keywords:** HIV, PMTCT, repeat testing, guideline implementation, maternal HIV testing, South Africa

## Abstract

**Introduction:**

The virtual elimination of mother‐to‐child transmission of HIV cannot be achieved without complete maternal HIV testing. The World Health Organization recommends that women in high HIV prevalent settings repeat HIV testing in the third trimester, and at delivery or directly thereafter. The Western Cape Province (South Africa) prevention of mother‐to‐child transmission (PMTCT) guidelines recommend a repeat maternal HIV test between 32 and 34 weeks gestation and at delivery in addition to testing at the first antenatal visit (ideally <20 weeks gestation). There are few published longitudinal studies on the uptake of initial and repeated maternal HIV testing programmes in sub‐Saharan Africa. We aimed to investigate the implementation of initial and repeat maternal HIV testing guidelines in Cape Town, South Africa.

**Methods:**

Between 2013 and 2016 we established an electronic PMTCT register that consolidated routine data from a primary healthcare facility and its secondary and tertiary referral sites in Cape Town. This provided a longitudinal record for each participant, from first antenatal visit to delivery. Utilizing these data, we conducted a retrospective analysis investigating the completeness of maternal HIV testing according to the PMTCT HIV testing guidelines in Cape Town, and predictors of complete testing, from 2014 to 2016.

**Results:**

Among 8558 enrolled pregnant women, 7213 (84%) were not known to be HIV positive at their first visit and thus eligible for HIV testing; 91% of them received ≥1 HIV test during pregnancy/delivery. Testing at the first visit was 98% among the 85% of women who attended antenatal care. Among women eligible to receive all three recommended HIV tests, only 11% achieved all three tests. Delivery HIV testing completion among all women without an HIV‐positive diagnosis was 23%. HIV prevalence at delivery was 21% and HIV incidence between first visit and delivery in those with ≥2 HIV tests was 0.2%. Women who enrolled after 2014 were more likely to receive the three recommended tests (aOR: 1.41; 95% CI: 1.10 to 1.81) and retest at delivery (aOR: 1.20; 95% CI: 1.05 to 1.39).

**Conclusions:**

Implementation of maternal HIV testing in Cape Town improved between 2014 and 2016 but major gaps remain, particularly at delivery.

## 1 Introduction

The implementation of policies recommending immediate lifelong triple antiretroviral therapy (ART) initiation for all HIV‐positive pregnant women (Option B+) has been shown to be effective in reducing mother‐to‐child transmission (MTCT) of HIV worldwide 1, 2. Women's access to these and other HIV‐related services in the prevention of MTCT (PMTCT) continuum relies on complete universal antenatal care (ANC) HIV testing, which is essential for the virtual elimination of MTCT of HIV 3, 4. It is estimated that failure to detect maternal HIV infection during ANC is responsible for up to 54% of MTCT in resource‐limited settings 5.

The World Health Organization (WHO) recommends that provider‐initiated HIV testing and counselling (PITC), forming part of ANC services, be offered to pregnant women at their first ANC visit (recommended at ≤12 weeks gestation) 6, 7. Not only will this enable the timely diagnosis of undiagnosed pregnant women living with HIV, it will also provide HIV‐negative women with guidance counselling and services to assist with the prevention of HIV acquisition and the opportunity to retest later during the pregnancy 8. In 2010, WHO recommended that women in areas with generalized HIV epidemics be re‐tested for HIV in either the third trimester, at delivery, or directly thereafter 9. In 2014, in the Western Cape (WC) Province, South Africa (SA), the *Western Cape Prevention of Mother‐to‐Child Transmission of HIV (PMTCT) Clinical Guidelines* recommended a repeat ANC HIV test between 32 and 34 weeks gestation and again at delivery, subsequent to testing at first ANC visit (recommended at <20 weeks gestation) 10. Retesting is important as pregnant women are more likely to acquire HIV infection compared to non‐pregnant women, and, if acutely infected, MTCT risk is increased in comparison with women who acquire HIV infection prior to pregnancy 11, 12, 13, 14, 15, 16. Incident maternal HIV infection was predicted to account for up to 34% of vertical infant infections in 2014 17. In addition, the rapid antibody assays may not accurately diagnose HIV during the window period (up to three months post acquisition) 13. In settings such as SA where >80% of deliveries occur in facilities 18, HIV testing at delivery provides a particular opportunity to test women with an unknown HIV status who do not attend ANC 19, 20.

Across sub‐Saharan Africa (SSA) initial antenatal HIV testing proportions range from 35% in Ethiopia to 99% in Malawi, but are mostly below the minimum 90% required to enable 90% of HIV‐positive pregnant women access to ART 4, 21, 22, 23, 24, 25. However, most studies were conducted before 2015 with very few reporting on post‐Option B+ policy implementation testing completion. Furthermore, these studies often use self‐report to determine HIV testing completion which could skew results due to reporting bias. In order to better understand the current (i.e. post‐Option B+) implementation and uptake of antenatal HIV testing in SSA studies utilizing recent routine data from ANC services are required. Additionally, since repeat HIV testing is provided sequentially throughout pregnancy, potentially involving multiple service providers, longitudinal individual maternal data are needed to accurately estimate repeat testing coverage. However, very few studies have collected such data and thus reports on coverage of repeated HIV testing in SSA are lacking 16, 26, 27, 28.

Utilizing prospectively collected, individual‐level, longitudinal patient data from a primary healthcare facility and its referral sites (secondary and tertiary) we aimed to investigate the implementation of and adherence to initial and repeat maternal HIV testing PMTCT guidelines in Cape Town, SA. We assessed: the coverage and timing of initial HIV testing during pregnancy, repeat HIV testing in the third trimester and at delivery, HIV prevalence and incidence among those tested, and the predictors of maternal HIV testing completion.

## 2 Methods

### 2.1 Study design

This study was a retrospective analysis conducted on antenatal maternal HIV testing data that were prospectively collected for the Closing the Gaps (CTG) study which has been described previously 29. Briefly, the CTG study established an integrated electronic PMTCT register (e‐register) that consolidated routine data from a primary care obstetric facility, Mitchells Plain Midwife Obstetric Unit (MPMOU), and its referral sites: Mitchells Plain District Hospital (MPDH), Mowbray Maternity Hospital (MMH) and Groote Schuur Hospital (GSH), within the urban Klipfontein/Mitchells Plain Sub‐Structure of the WC, SA.

### 2.2 Study setting

In the Western Cape, women with uncomplicated pregnancies can access antenatal care at Midwife Obstetric Units (MOUs) which can manage normal deliveries, or basic antenatal care (BANC) facilities which provide outpatient services only (these women will present to the MOU for delivery). MPMOU is within the Mitchells Plain Community Health Centre which is accessed by approximately 1.2 million people. Among community members, an estimated 24% are unemployed and 16% have lower grade education. At the time of the study MPMOU (primary care) provided ANC, including HIV testing, counselling and treatment, as well as midwife‐assisted uncomplicated vaginal delivery services 30. High‐risk pregnancies or those requiring advanced care were referred from MPMOU to a secondary or tertiary care facility during the antenatal or peri‐partum period. MPDH and MMH (secondary level) are equipped with operating theatres and neonatal intensive care unit (ICU) facilities respectively while GSH (tertiary level) has both adult and neonatal ICU facilities.

The 2015 WC antenatal HIV prevalence was reported to be 19% 31.

### 2.3 Study participants

In the CTG study, pregnant women, irrespective of HIV status, were passively enrolled at their first ANC visit at MPMOU, or alternatively, upon presentation for delivery if they had not received ANC. HIV tests (rapid antibody assay) were performed by community care workers as part of routine ANC services and by nurses in the labour ward of MPMOU and the labour/postnatal ward of the hospitals.

We included data from the CTG e‐register for all enrolled women who delivered at MPMOU or one of its referral sites, between 1 July 2014 and 31 December 2016, with a live or still birth pregnancy outcome. We excluded data from women who (a) presented for delivery having attended ANC at a BANC since we did not have access to complete longitudinal HIV testing data for these individuals or (b) for whom no pregnancy outcome could be found as we could not exclude pregnancy loss, in which case PMTCT testing guidelines would no longer be applicable.

### 2.4 Procedures and measurements (Box 1)

A longitudinal record, including HIV testing history from first antenatal visit (if attended) through to delivery, was extracted from the CTG e‐register for each woman. We categorized women as having attended ANC if they presented to a health facility >5 days before delivery. We defined early ANC attendance as ≤22 weeks gestation. We deemed all women eligible for an initial test if on their first visit, whether antenatally or at delivery, they were not already known to be HIV positive; and eligible for repeat testing if they had not had a prior positive antenatal HIV test. Tests were considered to be retests if women underwent an HIV test within the third trimester and/or at delivery following a prior negative HIV test. We considered women eligible for retesting in the third trimester if they delivered >35 weeks gestation. According to the guidelines there was no minimum time period between tests. The primary outcome was receipt of an HIV test (yes/no): (1) at first visit (ideally ≤22 weeks, but up to delivery); (2) in the third trimester as a repeat test; and (3) at delivery as a repeat test. The secondary outcome was HIV‐positive diagnoses (yes/no) at each test.

We calculated testing completion at:

- First ANC visit – in all eligible women who attended ANC.
- <28 weeks gestation (first test only) – in eligible women who attended their first ANC visit <28 weeks gestation.
- Third trimester (first and repeat testing assessed) – in eligible women who attended their first ANC visit before/during the third trimester (i.e. ≥28 weeks gestation); were not previously diagnosed HIV positive and delivered >35 weeks gestation.
- Delivery (±5 days) (first and repeat tests) – in eligible women irrespective of gestational age at delivery, ANC visit attendance or testing history.

**Figure 1:**
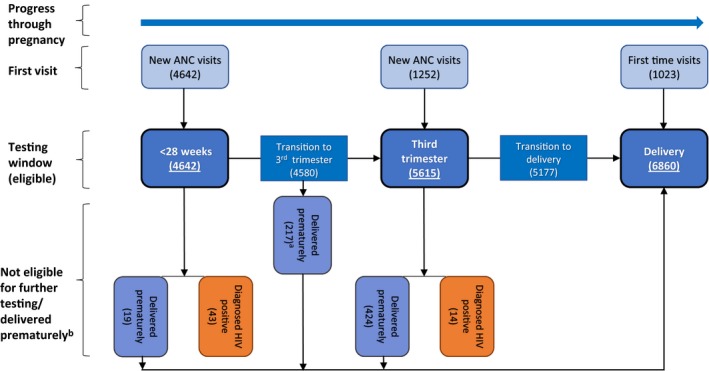
Flow chart illustrating the number of women eligible to be HIV tested within each testing point window. Only women for whom gestational age was available were included (n = 6917). ^a^Women who HIV tested <28 weeks gestation but delivered ≤35 gestation in the third trimester and therefore would not be eligible for repeat testing in the third trimester. ^b^Women who delivered prematurely were considered eligible for testing at delivery if not yet diagnosed HIV positive.

Women who tested at first ANC visit were also categorized as having tested <28 weeks gestation, in the third trimester and/or at delivery. Testing completion at each recommended window was calculated only among women eligible to be tested at that respective window (Figure 1; Table 2 – column 1). Data abstracted from the e‐register did not record ANC visits between the first visit and delivery unless a test was recorded. Eligibility for repeat testing in the third trimester was hence based on prior ANC attendance.

In addition, we calculated the testing completion restricted to the windows defined in the PMTCT guidelines (best practice) as follows:

- Eligible women must have attended ANC early (≤22 weeks gestation) and delivered >35 weeks gestation with a prior HIV‐negative status.
- Completion was defined as testing at three timepoints: ≤22 weeks gestation, in the third trimester, and at delivery.

Individuals diagnosed HIV positive during the study period were coded as “diagnosed at enrolment” if they tested HIV positive at their initial ANC test, or as “seroconverts” if they tested HIV‐positive after a previous negative antenatal HIV test. We categorized women as having an “uncertain HIV status at delivery” if they tested HIV negative at least once during ANC but had not received a test within three months prior to or at delivery; and having an “unknown HIV status at delivery” if they never tested during the current pregnancy. For women with two pregnancies within the study period, we included the pregnancy for which most data were available. Premature delivery was defined as delivery <37 weeks gestation.

Box 1 **Western Cape PMTCT guidelines 10, HIV testing eligibility and analysis explained.**

Western Cape PMTCT HIV testing guidelines^†^ 10:

▪ Test at first ANC visit (ideally <20 weeks gestation).
▪ Retest in third trimesters (32 to 34 weeks gestation or shortly thereafter).
▪ Retest at delivery.

Note: The guidelines do not specify a minimum time period between tests.

HIV testing eligibility defined as per the PMTCT guidelines^†^:

▪ Women who attended ≥ 1 ANC visit:
  ○ Eligible for ≥2 tests (minimum both first visit and delivery).
▪ Women with first ANC visit <28 weeks gestation:
  ○ Eligible for 3 tests (first visit, in third trimester, and delivery).
▪ Women with first ANC visit ≥ 28 weeks gestation:
  ○ Eligible for 2 tests (first visit and delivery).
▪ Women with no ANC attendance:
  ○ Eligible for 1 test (at delivery).

^†^The PMTCT guidelines assume best practice. Women do not attend ANC at recommended gestational ages and guidelines may be loosely implemented, for example, women may attended their first ANC visit and retest within the third trimester. We assessed HIV testing completeness as follows:

1. Testing completion at first visit at any timepoint (i.e. < or ≥28 weeks gestation, or at delivery).
2. Longitudinal testing completion throughout pregnancy, according to gestational age – (<28 weeks gestation, ≥28 weeks, at delivery).

▪ Longitudinal testing was assessed with both relaxed (Table 2) and strict (Table 3) eligibility criteria for retesting:
  ○ Relaxed – women could retest in third trimester and/or at delivery regardless of gestational age at first test.
  ○ Strict – women could only retest in third trimester and at delivery if they tested ≤22 weeks gestation and retested in the third trimester respectively.

### 2.5 Data analysis

Data analysis was carried out using Stata version 13.0 (Stata Corporation, College Station, Texas, USA). Differences between the characteristics of all participants, stratified by HIV status post delivery, were assessed using the two‐sample t‐test (normal distribution) or the Wilcoxon rank‐sum test (non‐normal distribution) for numerical data and the χ^2^ or Fishers Exact test for categorical data. A descriptive analysis (proportions) was used to assess HIV testing completion during pregnancy among participants for whom gestational age data was available. We used logistic regression to assess predictors of maternal HIV testing completeness. Multivariable models included several variables selected *a priori* as being possible risk factors (age at enrolment, gravidity, and year of enrolment) for the outcome. Additional variables were included by sequentially adding them based on univariable analysis of significance. Those that were either significantly associated with the outcome of interest after adjustment for other variables (*p* < 0.05) or that altered the odds ratios (ORs) for other variables in the model by ≥10% were retained 32.

### 2.6 Ethics

The University of Cape Town Human Research Ethics Committee and the Provincial Government of the Western Cape Department of Health Research Committee approved the study.

The CTG study was granted a waiver of informed consent for the e‐register as all data had already been collected routinely by health services. No participant recruitment was required.

## 3 Results

### 3.1 Participant characteristics

Between July 2014 and December 2016, 8558 women delivered at MPMOU or one of its referral sites with a live or still birth pregnancy outcome (Table 1). Within the study period, 153 women had two pregnancies of which only one pregnancy was included.

**Table 1:** Characteristics of 8558 participants stratified by HIV status post delivery. Characteristics at first visit (baseline) and delivery are reported^a^

Variable	HIV negative/uncertain^b^	HIV positive	*p*‐value^c^	Never tested
	n = 6557 (77%)	n = 1345 (16%)		n = 656 (8%)
Baseline characteristics				
Age (years): n (%)				
13 to < 16	76 (1)	3 (<1)	<0.001	6 (1)
16 to < 21	1074 (16)	75 (6)		111 (17)
21 to <31	3789 (58)	771 (57)		415 (63)
31 to <41	1506 (23)	481 (36)		116 (18)
41 to <47	103 (2)	15 (1)		8 (1)
Median (IQR)	26 (22 to 31)	29 (25 to 33)	<0.001	26 (22 to 30)
Gravidity: n (%)				
1	2019 (31)	236 (17)	<0.001	171 (26)
2	1922 (29)	475 (35)		188 (29)
3	1312 (20)	373 (28)		121 (18)
≥4	1197 (18)	226 (17)		167 (26)
Missing	107 (2)	35 (3)		9 (1)
Enrolment year: n (%)				
2014	2365 (36)	497 (37)	0.54	313 (48)
2015/2016	4192 (64)	848 (63)		343 (52)
Gestational age at first ANC visit (weeks): n (%)				
≤12	926 (14)	97 (7)	<0.001	4 (<1)
>12 to 20	1930 (29)	331 (25)		16 (2)
>20 to <28	1708 (26)	352 (26)		9 (1)
≥28	1211 (18)	252 (19)		30 (5)
No ANC	477 (7)	249 (19)		593 (90)
Missing	305 (5)	64 (5)		4 (1)
Median (IQR)	21 (15 to 29)	24 (18 to 35)	<0.001	40 (40–40)
Delivery characteristics				
Delivery facility: n (%)				
MPMOU	2648 (40)	551 (41)	0.09	422 (64)
MPDH	2264 (35)	485 (36)		160 (24)
MMH	1164 (18)	231 (17)		63 (10)
GSH	460 (7)	78 (6)		11 (2)
Missing	21 (<1)	0 (0)		0 (0)
Premature deliveries: n (%)				
Yes	607 (9)	110 (8)	0.46	48 (7)
No	5617 (86)	1166 (87)		7361 (88)
Missing	333 (5)	69 (5)		30 (5)
Birth outcome: n (%)				
Alive	6468 (99)	1320 (98)	0.17	643 (98)
Stillborn	89 (1)	25 (2)		13 (2)
HIV status at delivery: n (%)				
Known HIV positive before enrolment	–	1277 (95)	–	–
Diagnosed HIV‐positive at first visit	–	59 (4)		–
Seroconverts	–	9 (<1)		–
HIV negative	5092 (78)	–		–
Previously HIV negative^b^	1465 (22)	–		–
Not tested	–	–		656 (100)

ANC, antenatal care; GSH, Groote Schuur Hospital; IQR, Interquartile range; MMH, Mowbray Maternity Hospital; MPDH, Mitchells Plain District Hospital; MPMOU, Mitchells Plain Midwife Obstetric Unit; n, number of participants.

a Values are rounded to the nearest whole number and proportions do not always add up to 100%/variable;

b Individuals that were last tested during antenatal care ≥3 months before delivery and therefore have an uncertain HIV status at delivery;

c *p*‐values are reporting the significance of the difference between HIV negative/uncertain & HIV positive.

HIV‐negative/uncertain versus HIV‐positive women were more likely to attend ANC (93% vs. 81%; *p* < 0.001) and presented earlier for their first ANC visit (median gestation (IQR): 21 (15‐29) vs. 24 (18‐35) weeks; *p* < 0.001) (Table 1). At delivery, HIV status was confirmed in 75% of the entire cohort of whom 21% were positive. Among HIV‐positive women, 95% (n = 1277) were known positive at first ANC visit, 4% (n = 59) were newly diagnosed at the first ANC test and 0.7% (n = 9) seroconverted during pregnancy after an initial negative HIV test. Of the women who tested HIV‐negative antenatally, 22% had not tested within three months of delivery/at delivery (an uncertain HIV status). Among all women, 8% did not test during pregnancy/delivery at all. In total, 31% of all women who had not been diagnosed HIV‐positive before delivery had an unconfirmed HIV status (i.e. either uncertain or not tested at all during ANC) after delivery.

### 3.2 HIV testing completeness by gestational age and HIV incidence

Among all women for whom gestational age data were available (n = 6917), 85% attended ANC at MPMOU >5 days before delivery with 98% (95% CI: 97 to 98) HIV testing completeness at first visit (Table 2). Among HIV‐negative/unknown women who tested <28 weeks gestation, 0.9% (95% CI: 0.6 to 1.2) (43/4642) were newly diagnosed HIV positive at the first ANC visit. Among the 4296 (77%) of women who had previously tested HIV negative during their current pregnancy (at any time), 62% (95% CI: 61 to 64) were retested during the third trimester and 0.2% (95% CI: 0.1 to 0.4) (6/2666) seroconverted.

**Table 2:** HIV testing during pregnancy for women for whom gestational age data were available (n = 6917)

Testing window	All women eligible for testing^a^		Women previously untested in current pregnancy (initial testing)					Women previously tested in current pregnancy (repeat testing)		
	n	% (95% CI) tested	New visits		Previously attended ANC		% (95% CI) positive at first test^c^	n (%)	% (95% CI) tested	% (95% CI) positive at repeat test^d^
			n (%)	% (95% CI) tested	n (%)	% (95% CI) tested				
First ANC visit^b^	5894	98 (97 to 98)	5894 (100)	98 (97 to 98)	–	–	0.9 (0.6 to 1.2)	–	–	–
<28 weeks	4642	99 (98 to 99)	4642 (100)	99 (98 to 99)	–	–	0.9 (0.6 to 1.2)	–	–	–
Third trimester	5615	70 (68 to 71)	1252 (22)	97 (96 to 98)	67 (1)	33 (22 to 34)	0.6 (0.2 to 1.3)	4296 (77)	62 (61 to 64)^e^	0.2 (0.1 to 0.4)
Delivery	6860	23 (22 to 24)	1023 (15)	45 (41 to 48)	82 (1)	28 (17 to 39)	1.5 (0.5 to 3)	5755 (84)	19 (18 to 20)	0.3 (0.1 to 0.8)

ANC, antenatal care; n, number of participants.

a Testing eligibility (Figure 1): (a) First ANC visit: Women who attended ANC and were not known HIV positive (regardless of gestational age). (b) <28 weeks: Women who attended ANC <28 weeks gestation and were not known HIV positive. (c) Third trimester: Women who attended ANC before delivery, were not known HIV positive or diagnosed HIV positive <28 weeks gestation and *delivered >35 weeks gestation*·– only applicable to the eligibility for repeat testing. (d) Delivery: Women, regardless of ANC attendance status and gestational age, not diagnosed HIV positive;

b Data from first ANC visit are reported separately from longitudinal testing analysis;

c % positive was calculated as a proportion of women who tested for the first time: overall, <28 weeks gestation, in the third trimester or at delivery;

d % positive was calculated as a proportion of woman who received a repeat test in the third trimester or at delivery. In the third trimester the denominator included individuals who had their first ANC visit and had >1 test within the window;

e Completion of retesting in the third trimester does not distinguish between visit coverage and re‐testing coverage as we did not have a record of visit data separate from testing data.

Among women who did not attend any ANC (n = 1023), only 45% (95% CI: 21 to 48) received an HIV test at delivery, of whom 1.5% (95% CI: 0.5 to 3) (7/479) were newly diagnosed HIV positive. Of those women who had tested earlier in pregnancy (any number of tests at any stage), 19% (95% CI: 18 to 20) retested at delivery, of whom three women (0.3%; 95% CI: 0.1 to 0.8) had seroconverted. The highest delivery HIV testing completion was at the two secondary level facilities (MPDH: 32% and MMH: 21%) (Figure S1). Among women who attended ANC ≤22 weeks gestation, delivered >35 weeks gestation and were not diagnosed HIV positive before delivery (i.e. eligible for all three recommended tests) (n = 3177), 11% (95% CI: 10 to 12) received HIV tests in all three windows (Table 3). Of the 5% of women who were diagnosed HIV positive during this pregnancy, 64% were diagnosed at their first test before the third trimester and 9% were seroconversions during the third trimester (i.e. 28 weeks to delivery) (Figure S2). Among the 61% of women who attended ANC and had >1 HIV test, 0.2% (95% CI:0.1 to 0.5) seroconverted.

**Table 3:** Longitudinal HIV testing completion proportions among women with known gestational age who were eligible to receive all three recommended tests

Testing window	Women eligible: n^a^	Women tested: n (%: 95% CI)
≤22 weeks	3408	3358 (99: 98 to 99)
Third trimester	3153	2027 (64: 63 to 66)
Delivery	2024	343 (17: 15 to 19)
All three tests^b^	3177	343 (11: 10 to 12)

a Women are eligible for testing as follows: (a) ≤22 weeks: Women who attended ANC and were not known HIV positive). (b) Third trimester: Women who HIV tested ≤22 weeks gestation, were not known HIV positive or diagnosed HIV positive <28 weeks gestation and delivered >35 weeks gestation. (c) Delivery: Women who HIV tested ≤22 weeks and in the third trimester, delivered >35 weeks gestation, were not diagnosed HIV positive;

b All three tests eligibility includes women that attended ANC≤22 weeks gestation, were not diagnosed HIV positive before delivery and delivered >35 weeks gestation.

### 3.3 Predictors of maternal HIV testing missed opportunities

Among all eligible women (i.e. women not known HIV positive or diagnosed HIV positive at the first test), 91% had ≥1 HIV tests during pregnancy/delivery. In multivariable analysis, late gestational age at first ANC visit (aOR: 0.73; 95% CI: 0.71 to 0.75, per additional week gestation) was associated with being less likely to test for HIV at all (Table 4). Enrolment in 2015/2016 versus 2014 (aOR: 1.85; 95% CI: 1.51 to 2.29) was associated with receiving at least one HIV test.

Among women who attended ANC ≤22 weeks gestation, did not test positive for HIV before delivery and delivered >35 weeks gestation (i.e. eligible for all three HIV tests), 11% received all three of the recommended tests (Table 3). The following factors were associated with being more likely to receive all three recommended tests: enrolment in 2015/2016 vs. 2014 (aOR: 1.41; 95% CI: 1.10 to 1.81); and delivering at MPDH (aOR: 10.44; 95% CI: 6.97 to 15.67); or MMH (aOR: 5.17; 95% CI: 3.22 to 8.32); versus MPMOU (Table 4).

**Table 4:** Univariable and multivariable factors associated with completion of A. initial HIV testing, B. receiving the three recommended tests among eligible women, C. testing at delivery among women who did not attend ANC and D. retesting at delivery among eligible women

Variable	A. Initial HIV testing (n = 6886)		B. Receiving three recommended tests^a^ (n = 3187)		C. Testing at delivery (no ANC) (n = 1016)		D. Retesting at delivery (n = 5458)	
	OR (95% CI)	aOR (95% CI)	OR (95% CI)	aOR (95% CI)	OR (95% CI)	aOR (95% CI)	OR (95% CI)	aOR (95% CI)
Age at first visit (years)	1.02 (1.01; 1.03)	1.02 (0.99; 1.05)	0.99 (0.97; 1.01)	1.00 (0.97; 1.02)	1.05 (1.03; 1.08)	1.01 (0.98; 1.04)	0.99 (0.98; 1.00)	0.99 (0.97; 1.00)
Gestational age at first visit (weeks)	0.73 (0.72; 0.76)	0.73 (0.71; 0.75)	1.01 (0.98; 1.03)	b	b	b	1.00 (0.99; 1.01)	b
Gravidity: 1	Ref	Ref	Ref	Ref	Ref	Ref	Ref	Ref
2	0.87 (0.70; 1.08)	1.01 (0.75; 1.36)	0.77 (0.58; 1.01)	0.86 (0.64; 1.17)	1.37 (0.94; 1.99)	1.29 (0.85; 1.95)	0.73 (0.62; 0.87)	0.82 (0.67; 1.00)
3	0.92 (0.72; 1.17)	1.23 (0.86; 1.76)	0.71 (0.51; 0.98)	0.85 (0.58; 1.24)	1.82 (1.27; 2.70)	1.51 (0.95; 2.40)	0.73 (0.61; 0.89)	0.90 (0.71; 1.14)
≥4	0.60 (0.48; 0.76)	1.16 (0.79; 1.72)	0.78 (0.55; 1.10)	0.90 (0.58; 1.40)	2.72 (1.91; 3.90)	2.05 (1.24; 3.38)	0.80 (0.66; 0.98)	0.98 (0.74; 1.29)
Premature delivery: Yes	Ref	b	Ref	b	Ref	Ref	Ref	b
No	0.77 (0.57; 1.05)	b	1.01 (0.62; 1.64)	b	0.60 (0.39; 0.93)	0.59 (0.34; 1.05)	0.87 (0.69; 1.08)	b
Mode of delivery: Vaginal	Ref	b	Ref	b	Ref	Ref	Ref	Ref
Assisted	2.08 (0.50; 8.64)	b	2.42 (0.81; 7.22)	b	1.93 (0.32; 11.61)	4.59 (0.68; 31.13)	1.61 (0.68; 3.78)	0.85 (0.35; 2.07)
Born before arrival	0.26 (0.18; 0.37)	b	b	b	1.74 (1.16; 2.63)	1.28 (0.83; 1.97)	0.42 (0.13; 1.37)	0.74 (0.22; 2.57)
Caesarean section	2.31 (1.83; 2.91)	b	2.04 (1.62; 2.57)	b	0.73 (0.48; 1.12)	1.21 (0.70; 2.07)	1.64 (1.43; 1.90)	0.84 (0.71; 0.99)
Outcome: Alive	Ref	b	Ref	b	Ref	Ref	Ref	b
Still born	0.68 (0.38; 1.22)	b	0.76 (0.10; 5.87)	b	2.12 (1.03; 4.39)	1.40 (0.61; 3.19)	0.52 (0.18; 1.48)	b
Year of enrolment: 2014	Ref	Ref	Ref	Ref	Ref	Ref	Ref	Ref
2015/2016	1.58 (1.35; 1.86)	1.85 (1.51; 2.29)	1.32 (1.03; 1.68)	1.41 (1.10; 1.81)	2.82 (2.15; 3.70)	2.49 (1.88; 3.30)	1.20 (1.05; 1.39)	1.33 (1.15; 1.55)
Delivery facility: MPMOU	Ref	Ref	Ref	Ref	Ref	Ref	Ref	Ref
MPDH	2.25 (1.86; 2.72)	0.86 (0.66; 1.11)	10.45 (6.97; 15.67)	10.44 (6.95; 15.68)	0.51 (0.37; 0.71)	0.60 (0.40; 0.92)	7.12 (5.87; 8.63)	8.18 (6.64; 10.07)
MMH	2.96 (2.25; 3.89)	0.71 (0.50; 1.00)	5.11 (3.19; 8.19)	5.17 (3.22; 8.32)	0.80 (0.50; 1.28)	0.62 (0.33; 1.14)	3.65 (2.91; 4.58)	4.14 (3.21; 5.34)
GSH	6.64 (1.62; 12.18)	0.52 (0.22; 1.20)	0.73 (0.22; 2.42)	0.74 (0.22; 2.46)	1.52 (0.48; 4.83)	1.40 (0.39; 5.02)	1.66 (1.10; 2.52)	1.96 (1.23; 3.13)
Prior ANC testing: Tested ≤22 weeks and third trimester	b	b	b	b	b	b	Ref	Ref
Tested ≤22 weeks only	b	b	b	b	b	b	1.33 (1.13; 1.55)	1.40 (1.18; 1.65)
Tested third trimester only	b	b	b	b	b	b	1.29 (1.09; 1.54)	1.55 (1.29; 1.87)

ANC, antenatal care; aOR, Adjusted odds ratio; CI, confidence interval; GSH, Groote Schuur Hospital; MMH, Mowbray Maternity Hospital; MPDH, Mitchells Plain District Hospital; MPMOU, Mitchells Plain Midwife Obstetric Unit; n, number of participants.

a Only women that attended ANC ≤22 weeks gestation, were not diagnosed HIV positive before delivery and delivered >35 weeks gestation were eligible for all three tests;

b Not included in the model.

An HIV test at delivery was recommended for all women. Among those eligible for HIV retesting at delivery (i.e. previous HIV‐negative test in the current pregnancy), 19% tested (Table 2). In multivariable analysis the following factors were associated with retesting at delivery: enrolment in 2015/2016 versus 2014 (aOR: 1.33; 95% CI: 1.15 to 1.55); having only tested ≤22 weeks gestation (aOR: 1.40; 95% CI: 1.18 to 1.65), or having only tested in the third trimester (aOR: 1.55; 95CI: 1.29 to 1.87) vs. having tested both ≤22 weeks gestation and in the third trimester; and delivering at MPDH (aOR: 8.18; 95% CI: 6.64 to 10.07), MMH (aOR: 4.41; 95% CI: 3.21 to 5.34), or GSH (aOR: 1.96; 95CI: 1.23 to 3.13) versus MPMOU (Table 4). Having a caesarean section versus vaginal delivery was associated with being less likely to test at delivery (aOR: 0.84; 95% CI: 0.71 to 0.99).

Among eligible women (i.e. not known HIV positive) who did not attend ANC, 45% tested at delivery (Table 2). Those who enrolled in 2015/2016 versus 2014 were more likely to test at delivery (aOR: 2.49; CI: 1.88 to 3.30) (Table 4).

## 4 Discussion

This is, to the best of our knowledge, one of the first longitudinal studies of the implementation of maternal HIV testing guidelines in a routine setting in SA. The majority of women eligible for HIV testing tested at least once (91%) during pregnancy or at the time of delivery. Although testing completion at first ANC visit was high (98%), women attended ANC late with only 39% doing so <20 weeks gestation. While HIV testing implementation improved over time, with increased completion in the second year after guideline change, there were substantial missed testing opportunities with a third of women not testing within three months of or at delivery and only 11% of eligible women being tested in all three recommended windows.

The high proportion of women receiving ≥1 HIV test in our study is encouraging; however, it falls short of a) the global PMTCT target of having over 95% of pregnant women aware of their HIV status and b) the >95% “uptake of antenatal HIV testing” rate reported for SA between 2010 to 2012 15. This observed difference may be due to our use of operational data from a single referral chain of health facilities in one area of Cape Town, including testing completeness in women presenting at delivery with no prior ANC, versus the use of nationwide aggregate antenatal survey data. This is underscored by earlier gestational age at first ANC visit (per additional week gestation) being a significant predictor of initial HIV testing in pregnancy in our analysis, concurring with results from Zimbabwe 33. These results suggest a need for additional interventions to improve the implementation of initial HIV testing among women who present to ANC late in pregnancy or not at all.

In SA attending ANC before 20 weeks gestational age, regardless of HIV status, is a PMTCT‐related indicator 15. It is also recommended in national and WC guidelines that women are HIV tested at their first visit 8, 10. Reassuringly, in our study the implementation of HIV testing at first ANC visit was excellent, but unfortunately many women attended ANC late i.e. after 20 weeks gestation. Furthermore, a high proportion of women presented at delivery with unknown HIV status and having not attended ANC. Of the women who did not attend ANC and tested at delivery, 1.5% tested HIV positive which may be an underestimate as less than half of women who did not attend ANC received an HIV test at delivery. These women and their infants left the facility with unknown HIV and HIV exposure status respectively. Untested women may have had a different HIV risk profile to tested women.

Reassuringly, implementation of maternal HIV testing in our setting improved over time following the adoption of the updated PMTCT guidelines in 2014, similar to results from Kenya 26. Women were more likely to complete testing if they enrolled in 2015/2016 as opposed to in 2014. Notwithstanding, we observed several missed repeat testing opportunities; a third of women had an unconfirmed HIV status after delivery as they had either never tested or last tested ≥3 months before delivery. This is lower than that observed in Kenya 26. Only a small proportion of eligible women received all three recommended HIV tests. The data suggest that poor implementation of delivery testing was responsible for the bulk of missed opportunities.

At delivery, women were more likely to HIV retest at either of the three referral hospitals (MPDH/MMH/GSH) versus MPMOU. At these facilities there were dedicated PMTCT nurses designated to do HIV testing in contrast to the MOU where HIV testing fell within the remit of all midwives. Women were often tested in the labour ward upon arrival, but otherwise had other opportunities to test in the postnatal ward during an admission of a few days. In primary care, women are often discharged within six to eight hours of an uncomplicated birth.

HIV prevalence among women with a known HIV status at delivery was similar to that reported for the WC 31. Of the HIV‐positive women the vast majority were known positive at enrolment indicating the maturity of the HIV programme with high overall testing coverage. It is also reassuring that the first of the 90‐90‐90 targets 15, that 90% of HIV‐positive people know their status, has been met. It should however be noted that this proportion may be slightly overestimated due to missed positive diagnoses among women that never tested. Furthermore, awareness of HIV status does not guarantee that women have initiated and adhered to ART. Further studies are required to assess viral load suppression among known positive women. The overall HIV incidence among women that initially tested HIV negative was estimated to be substantially lower than that previously reported in SA and SSA (3 to 4%), but similar to that of non‐African countries (0.3%) 12, 34. While our study may substantially underestimate incidence due to the low completeness of repeat HIV testing, assessment of the most cost‐effective number and timing of maternal HIV tests in different incidence settings, and feasibility of implementation, should be considered for future studies.

### 4.1 Strengths and limitations

The use of individual‐level longitudinal data allowed for a more accurate assessment of testing completion, based on each woman's progress through the PMTCT continuum as opposed to using aggregate data. The use of longitudinal data also enabled the description of (a) the timing of HIV‐positive diagnoses and (b) HIV incidence estimates over the course of pregnancy. Point‐of‐care HIV testing data are not routinely digitized in SA so to our knowledge these data are rare. We had a large sample size which included antenatal and delivery HIV testing data from primary through tertiary care facilities. Positively, our study demonstrated the real‐world implementation of PMTCT testing guidelines; however, we were dependent on the quality of routine operational data and could not account for missing data. Furthermore, qualitative data were not routinely collected, and we could not explore qualitative factors associated with testing completion. At visits other than first ANC visit and delivery, we did not have a record of visit data separate from testing data and therefore could not distinguish between visit coverage and re‐testing coverage in the third trimester. It is therefore possible that retesting coverage in the third trimester is reflective of poor ANC attendance as opposed to poor implementation of PMTCT guidelines. Although we excluded women who attended any documented ANC visits at facilities outside MPMOU, we were unable to determine whether women had received point‐of‐care HIV testing at facilities not included in the study, or outside the WC province. The subset of women without recorded pregnancy outcomes may have had systematically different testing outcomes to those for whom outcomes were recorded and their exclusion from the dataset may therefore have introduced selection bias into the study results, thereby slightly overestimating testing completeness. Although the results of this study may be externally valid to the WC, HIV testing is context‐specific, and results should be treated with caution when generalizing to other settings.

## 5 Conclusions

The results of this study illustrate that although there has been maturation of maternal HIV testing within the PMTCT programme over time, gaps remain in late pregnancy follow‐up testing, particularly at delivery. Interventions are required at facility level to improve delivery HIV testing among women starting ANC late/not attending ANC and those that have high‐risk pregnancies in order to limit the transition of undiagnosed HIV‐positive women to the postnatal period without access to lifelong ART, feeding support and infant post‐exposure prophylaxis. Additional research to assess (a) the viral suppression among known HIV‐positive women and (b) the cost‐effectiveness of PMTCT testing guideline implementation is required.

## Competing interest

The authors have no Competing interest to declare.

## Authors' contributions

MAD, EK, AB, MK and MO conceptualized and designed the research study, contributed to operational needs and clinical oversight when required. EK, JE, VT and MAD contributed to data management. EK and MAD contributed to data analysis. SDB cleaned and analysed the data and prepared the manuscript. MAD, EK and JE critically reviewed the manuscript. All authors have read and approved the final manuscript.

## Supporting information


**Figure S1.** Delivery HIV testing completion at respective delivery facilities.

**Figure S2.** Diagnoses among HIV‐positive women^a^ (n = 1344).
