# Supplementary figures and images for: A longitudinal analysis of the completeness of maternal HIV testing, including repeat testing in Cape Town, South Africa
Source: J Int AIDS Soc. 2020 Jan 29;23(1):e25441. doi: 10.1002/jia2.25441 (PMC6989397; doi:10.1002/jia2.25441)

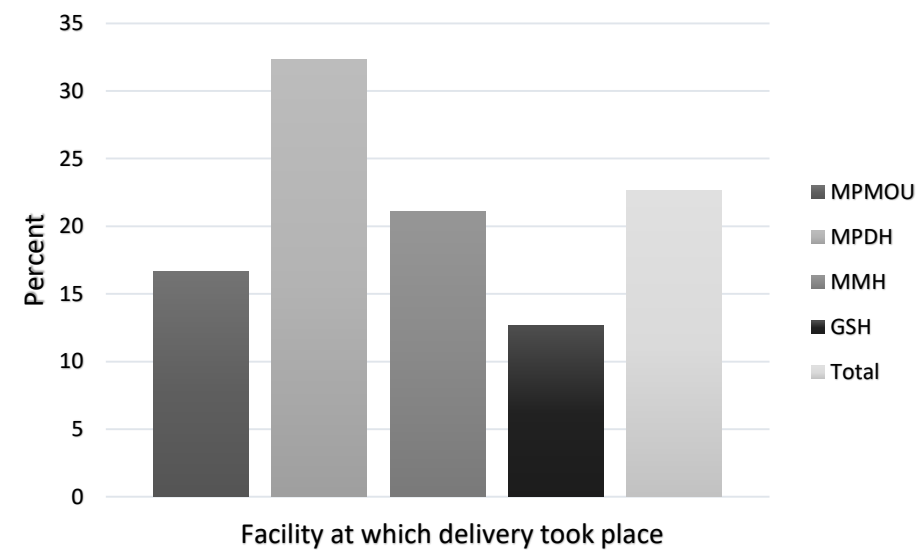

---

Supplement: Supplementary file 1 — Figure S1. Delivery HIV testing completion at respective delivery facilities. [file JIA2-23-e25441-s001.pdf]

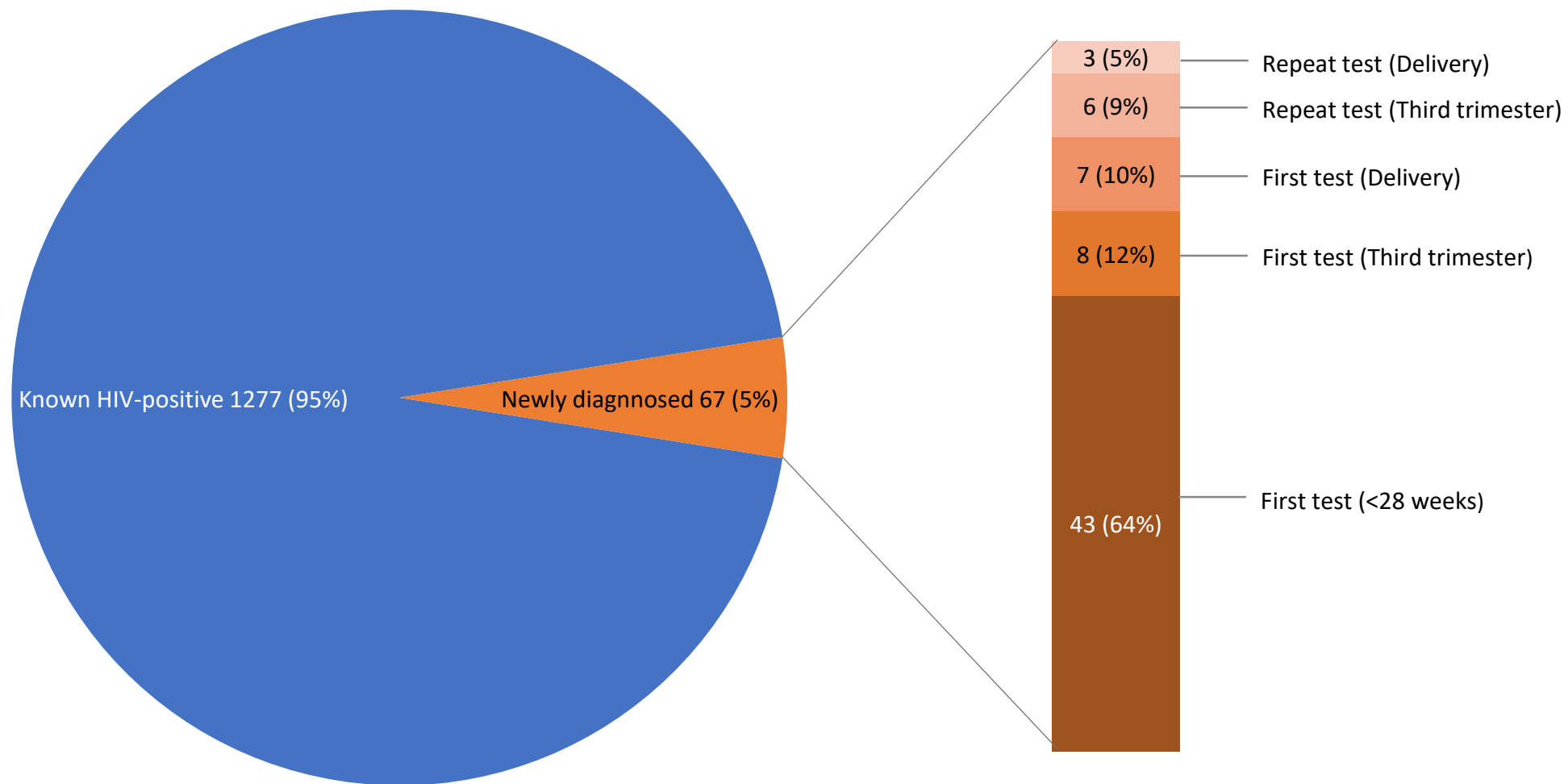

---

Supplement: Supplementary file 2 — Figure S2. Diagnoses among HIV‐positive womena (n = 1344). [file JIA2-23-e25441-s002.pdf]
